# Design and Study of a Reflector-Separated Light Dispersion-Compensated 3D Microscopy System

**DOI:** 10.3390/s23094516

**Published:** 2023-05-06

**Authors:** Hui Li, Xin Tan, Qingbin Jiao, Yuhang Li, Siqi Liu, Jian Pei, Jiahang Zhang, Wei Zhang, Liang Xu

**Affiliations:** 1 Changchun Institute of Optics, Fine Mechanics and Physics, Chinese Academy of Sciences, Beijing 130033, Chinatanxin@ciomp.ac.cn (X.T.);; 2 University of Chinese Academy of Sciences, Beijing 100049, China

**Keywords:** tomographic microscopy system, secondary-phase grating, dispersion compensation, optical design

## Abstract

The secondary-phase grating-based tomographic microscopy system, which is widely used in the biological and life sciences, can observe all the sample multilayer image information simultaneously because it has multifocal points. However, chromatic aberration exists in the grating diffraction, which seriously affects the observation of the image. To correct the chromatic aberration of the tomographic microscope system, this paper proposes a system that adopts blazed gratings and angle-variable reflectors as chromatic aberration correction devices according to the principle of dispersion compensation and Fourier phase-shift theory. A reflector-separated light dispersion-compensated 3D microscopy system is presented to achieve chromatic aberration correction while solving the problem of multilayer image overlap. The theoretical verification and optical design of the system were completed using ZEMAX software. The results show that the proposed system reduced the chromatic aberration of ordinary tomographic microscopy systems by more than 90%, retaining more wavelengths of light information. In addition, the system had a relatively wide range in the color difference compensation element installation position, reducing the difficulty of dispersion compensation element installation. Overall, the results indicate that the proposed system is effective in reducing chromatic aberration in grating diffraction.

## 1. Introduction

In recent years, tomographic microscopy has been widely used in the biological and life sciences. It is commonly used for 3D structure detection of biological cells and for the observation of the life activities of tiny living organisms [1,2,3,4,5,6]. Optical microscopes allow for the observation of transparent objects but usually provide only superficial images, which do not permit the observation of the internal structure of the object. The 3D microscope was developed based on the principle of secondary-phase grating spectroscopy [7,8,9,10,11,12], which enables real-time acquisition of the multilayer image information of biological samples in a single observation [13,14], eliminating the need to observe multiple layers of cell sample sections. It greatly improves the acquisition and observation efficiency. Therefore, secondary-phase gratings, also known as tomographic gratings, are the core devices in optical tomographic microscopy systems. However, chromatic aberration exists in tomographic gratings under broadband illumination. Multiple wavelengths overlapping each other can cover the details of the image and affect the judgment of the image details [9].

To obtain clear color images of tomographic microscopy, Lin [15] et al. used band-pass filters, while Yuan [16] et al. used a single-wavelength laser outputting narrow-band light to obtain tomographic images in the narrow-band range. Moreover, Zhu, as well as Tao et al., used a super surface to phase modulate the diffracted light to achieve single-wavelength tomographic imaging by using the difference in light wavelength [17]. Feng et al. used a prism grating to create a “pre-dispersion” [18,19,20] opposite to the dispersion of the tomographic grating to offset the chromatic aberration. Further, Abrahamsson et al. used a combination of a chromaticity-corrected grating and a refractive prism [21,22,23]. The dispersion of the prism and tomographic grating is compensated for by adjusting the grating period of the chromaticity-corrected grating.

However, current chromatic aberration correction methods for tomographic gratings have the following problems: narrow-band imaging avoids chromatic aberrations but does not yield single-layer color images [24], which causes the loss of most wavelength information. The chromatic aberration compensation method can obtain color tomographic images [25], but the chromatic aberration compensation structure is difficult to install and adjust. Moreover, the material refractive index errors existing in multiple chromatic aberration correction elements can lead to chromatic aberration residuals. Therefore, in this paper, the chromatic aberration of the tomographic grating is reduced by using blazed grating with the same substrate based on the principle of dispersion compensation. To solve the multilayer image overlap problem in the chromatic aberration correction method, we establish a tomographic diffraction-level separated-light model. Three reflectors with an adjustable tilt angle are used instead of refractive prisms to separate light and solve the problem of image overlap. The theoretical verification and optical design of the reflector-separated light dispersion-compensated 3D microscopy system were completed by ZEMAX software.

## 2. Theoretical Design

### 2.1. Composition of Reflector-Separated Light Dispersion-Compensated 3D Microscopy System

**Figure 1:**
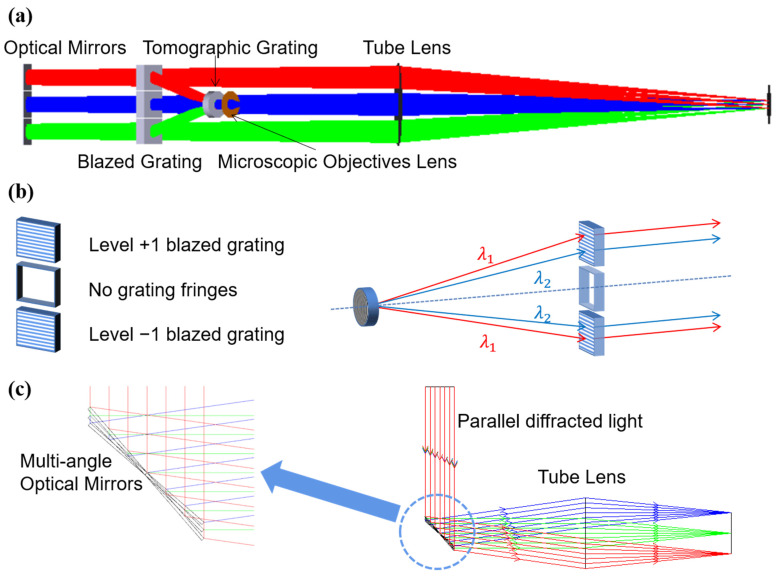
(**a**) Optical path model of the reflector−separated light dispersion−compensated 3D microscopy system; (**b**) schematic diagram of blazed grating chromatic aberration compensation; (**c**) schematic diagram of three reflectors separating light, where different colors represent different reflectors corresponding to the three tomographic diffraction levels.

The optical path model of the reflector-separated light dispersion-compensated 3D microscopy system is shown in Figure 1a. The whole system consists of a tomographic module, a reflector-separated light dispersion–compensation module, and an imaging module. The tomographic module includes a microscope objective and tomographic grating; the reflector-separated light dispersion–compensation module includes blazed grating and reflectors; and the imaging module includes a tube lens and charged–coupled device (CCD). The tomographic module has three different object-side focal planes. The light on these planes is separated in the form of parallel light according to diffraction levels −1, 0, and +1, and it then shines on the blazed grating. The tomographic grating is placed on the back focal plane of the objective lens, that is, the aperture stop. The blazed grating is composed of upper, middle, and lower parts, where the middle part corresponds to level 0 of the tomographic grating because there is no chromatic aberration in the tomographic grating level-0 diffraction, so there is no grating stripe in the middle part. The upper and lower parts are blazed gratings. Grating period $d_{2}$ is the same as the central period of tomographic grating $d_{1}$, and the blazed level is the opposite of the tomographic diffraction level. As shown in Figure 1b, the diffracted light from the tomographic grating with chromatic aberration is corrected to parallel light of different wavelengths that do not exactly coincide with each other, and the light of different wavelengths has the same diffraction angle to achieve chromatic aberration correction.

According to the Fourier phase-shift theorem, it is known that if the imaging module is added directly after the blazed grating, the light of different tomographic diffraction levels will be parallel. The images on the CCD will overlap, and it will be impossible to achieve a clear observation of each layer. Therefore, it is necessary to change the transmission direction of the beam of the three tomographic diffraction stages after the blazed grating. In this paper, three tilt-angle adjustable reflectors are used as the splitting devices for the tomographic diffraction beams, and the difference in diffraction angle is manufactured. The separation of the three tomographic images is finally realized on the CCD, and the optical principle is shown in Figure 1c. The three reflectors used are arranged in the same way as the previous blazed grating used for chromatic aberration correction. Each reflector corresponds to one diffracted beam output from the blazed grating, and the three mirrors are rotated co-axially when adjusting the angle. To avoid additional optical range differences between the three tomographic diffraction stages, we rotate the mirrors around the Y-axis only. In Figure 1c, the three reflectors overlap along the Y-axis direction. The direction of diffraction level light propagation can be controlled by adjusting the angle of reflectors. The angular difference between the three tomographic diffraction beams is controlled according to the focal length of the tube lens to separate the three tomographic images.

### 2.2. Theoretical Design and Parameter Selection of the Reflector-Separated Light Dispersion-Compensation Module

**Figure 2:**
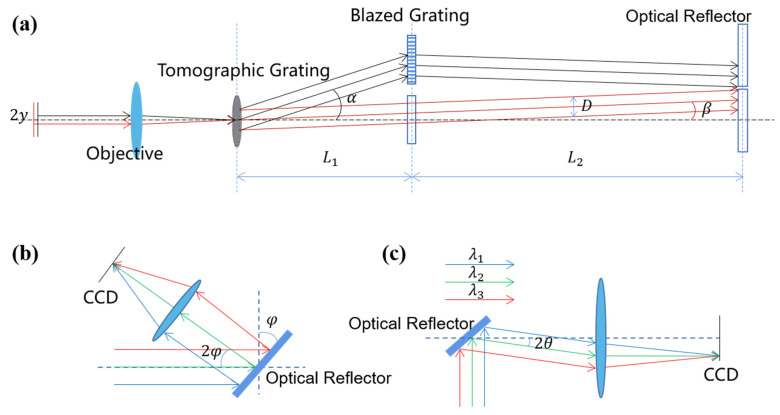
(**a**) Structure of the chromatic aberration correction part of the tomographic grating; (**b**) schematic diagram of the reflector-separated light; (**c**) structure of chromatic aberration correction of tomographic +1-level diffracted light.

Figure 2a shows the profile of the system from the object plane to the reflector plane, and only levels 0 and +1 are drawn to facilitate the calibration parameters. Figure 2b shows the profile from the reflector plane to the CCD plane at one diffraction level, and Figure 2c shows the effect of the reflector tilt on the image position on the CCD. In the case of levels +1 and 0, for example, the design process must ensure that the +1 and 0 diffracted light are separated in the plane of the reflector. If the level-+1 and level-0 diffracted light overlap on the reflector plane, the corresponding reflectors will overlap, the reflector will be unable to achieve separate regulation of the light angle of a single diffraction stage, and the image chromatic aberration of the overlapped part cannot be corrected. As shown in Figure 2a, let the maximum line field of view of the object side be 2y. The through-light aperture is D; the incident angle of the tomographic grating corresponding to the maximum line field of view is *β*, and the corresponding diffraction angle is α; the distance between the tomographic grating and the blazed grating plane is $L_{1}$; and the distance between the blazed grating plane and the reflector plane is $L_{2}$. The condition (1) that the two diffracted light beams do not coincide is expressed by Equation (1):

(1) $$H_{1}-H_{2}\geq D.$$

$H_{1}$ is the height of the primary ray in the plane of the reflector relative to the optical axis for the maximum line field of view on the object side.

(2) $$H_{1}=L_{1}tan\alpha -L_{2}tan\beta$$

$H_{2}$ is the height of the main ray of the object-side edge field of view of the tomographic grating level-0 diffracted light in the plane of the reflector relative to the optical axis.

(3) $$H_{2}=\left(L_{1}+L_{2}\right)tan\beta$$

A blazed grating corresponds to the beam of only one tomographic diffraction stage, and the location of the blazed grating must ensure that the three diffracted beams do not overlap. Thus, there is constraint 2:

(4) $$L_{1}\geq \frac{D}{tan\alpha }.$$

The +1-level diffracted beam aperture increases owing to the grating dispersion nonlinearity. Let the diffraction angle of the −1 field of view be α′ and the deformation variable be no larger than 10% of the original spot. Thus, there is constraint 3:

(5) $$L_{1}\left(tan\alpha^{\prime }-tan\alpha \right)\leq 0.1D.$$

The angle of inclination of the main reflector is *φ*, and the angle of deflection of the light is 2*φ*. In the design, it is necessary to ensure that the reflected light cannot overlap with the blazed grating, as shown in the figure. Thus, there is constraint 4:

(6) $$L_{2}\geq \frac{D}{tan2\varphi }.$$

The maximum number of etched line gratings made by wet etching should not exceed 500 L/mm. Tomographic grating is a variable-period grating. According to the variable relationship between the grating period and grating size, the number of etched lines in the center of tomographic grating is fixed at 400 L/mm, and the corresponding center period is 2.5 μm. By combining the above-derived equations with the existing microscope objective parameters, we give the parameters of the tomographic module and the reflector-separated light dispersion–compensation module, as shown in Table 1.

**Figure 3:**
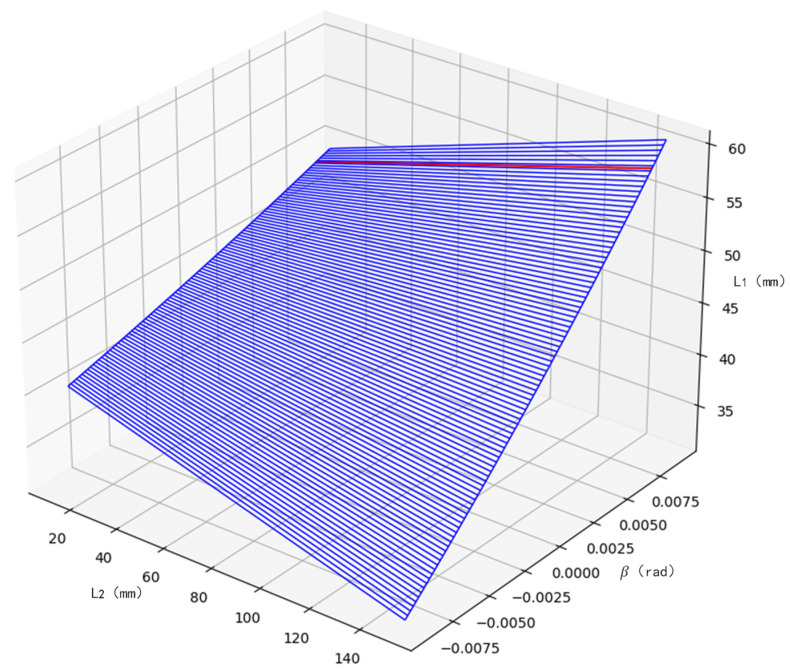
$L_{1},L_{2},\beta$ parameter constraint range map.

To satisfy the requirements of Equation (1), we plot the relationship between $L_{1},L_{2},$ and *β* by combining the parameters in Table 1 and constraints 1, 2, 3, and 4, as shown in Figure 3. Above the blue surface are the combinations of $L_{1},L_{2}$, and *β* that can be selected. Set the object-side field of view to 0.3 mm, corresponding to the *β* value of 0.008 and corresponding to the red curve in Figure 3. The range of values of $L_{1}$ and $L_{2}$ is derived from constraints 2, 3, and 4 as follows: 40 mm ≤ $L_{1}$ ≤ 62 mm, $L_{2}$ ≥ 12.5 mm. The priority in the parameter selection is to make $L_{1}$ as large as possible and to move $L_{2}$ away from the boundary, leaving a margin for installation. The final choice is 60 mm for $L_{1}$ and 50 mm for $L_{2}$.

### 2.3. Imaging Module Parameters Selection

From the parameters in Section 2.2, it can be roughly calculated that the aperture of the tube lens is 36 mm. However, the effective aperture of the existing tube lens in the laboratory is 24 mm, so it is necessary to design a tube lens to meet the conditions of use and correct the chromatic aberration of the tube lens. Then, the three tomographic images can be arranged sequentially on the detector without interfering with each other, and the image edges will be within the effective range of the detector. The tilt angle between the reflectors is *θ*, the magnification of the microscope is *γ*, and the effective size of the detector available in the laboratory is *Y*. The constraint is expressed by Equation (7):

(7) $$\frac{Y}{2}-y\gamma \geq f_{2}^{\prime }tan2\theta \geq 2y\gamma .$$

The technical parameters of the imaging module are shown in Table 2.

## 3. Results and Analysis

In this paper, the chromatic aberration of the tomographic grating is reduced by using blazed gratings with the same substrate according to the principle of dispersion compensation. The Fourier phase-shift theorem is used to solve the image coincidence problem by using three reflectors with adjustable tilt angles as the beam-splitting device of the dispersion compensation module. The theoretical verification and optical design of the reflector-separated light dispersion-compensated 3D microscopy system were carried out using ZEMAX software.

**Figure 4:**
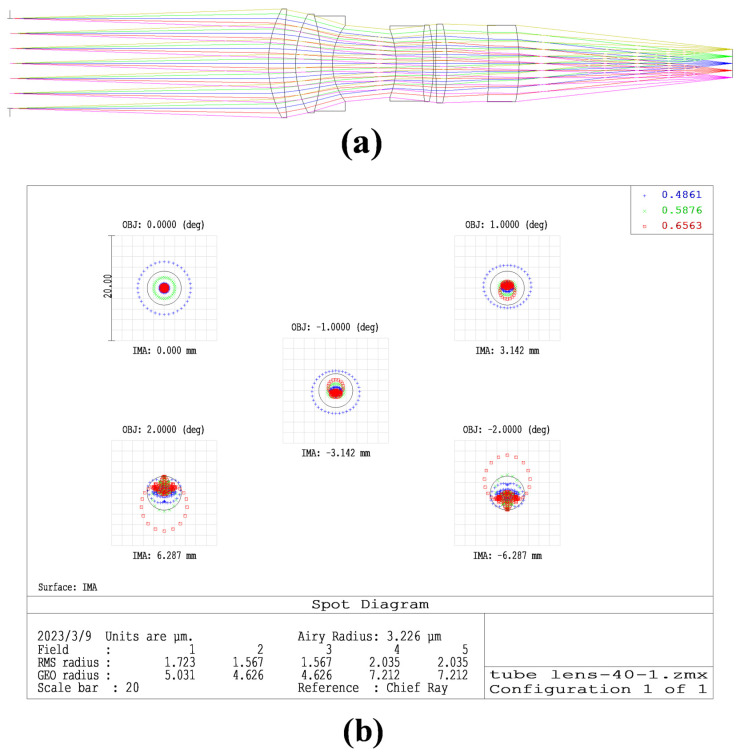

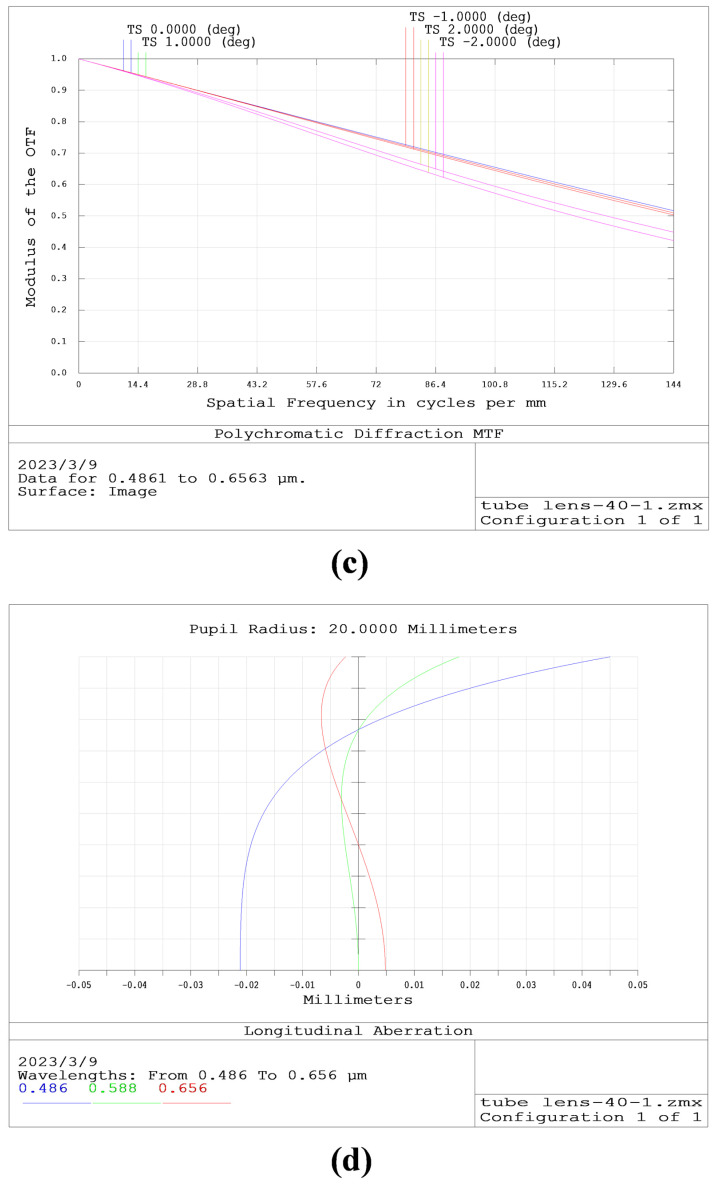
(**a**) Optimized optical structure of the barrel mirror; (**b**) spot diagram of the tube lens; (**c**) MTF curve of the tube lens; (**d**) chromatic aberration correction curve of the tube lens.

The tube lens requirements to design the image-space telecentric structure are in accordance with the parameters in Table 2 [26]. To simplify the design, we chose a double Gaussian objective as the original structure [27]. To meet the design requirements, we added a lens during the design process. The final optical structure and image quality evaluation are shown in Figure 4. The optical structure of the tube lens is in Supplementary Material S1.

The results show that the RMS radius of the optical structure of the tube lens is close to the Airy spot radius; that the MTF curve is greater than 0.4 at 144 lp/mm; that the entrance pupil of the tube lens is 40 mm; and that the through-light aperture is 48 mm, which meets the design index. Further, the chromatic aberration curves of F-light and C-light intersect at the 0.707 field of view, and the chromatic aberration is also corrected.

**Figure 5:**
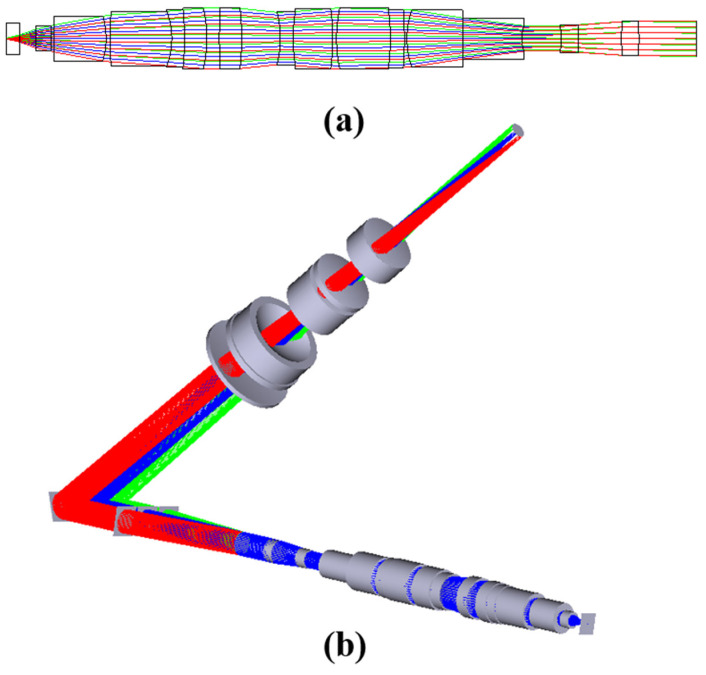

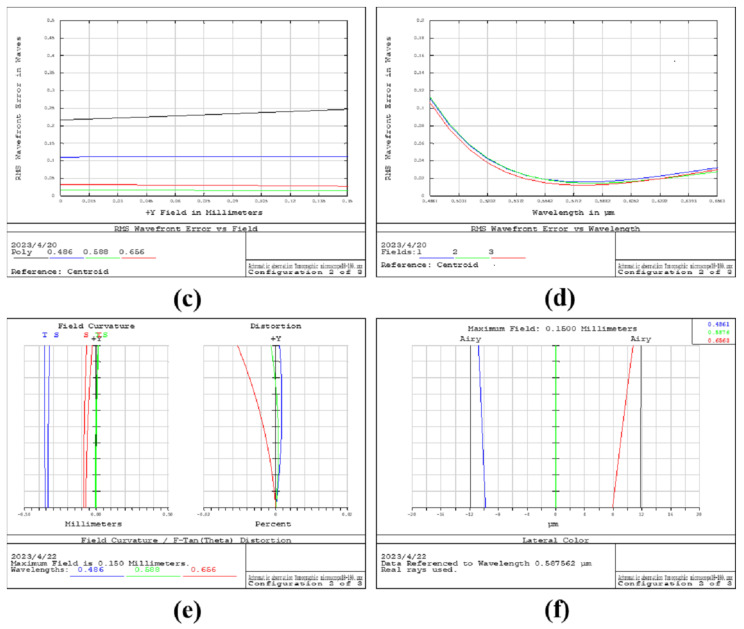
(**a**) Optical structure of the microscope objective; (**b**) optical structure of reflector−separated light dispersion−compensated tomographic microscopy system; (**c**) RMS vs. field−of−view diagram; (**d**) RMS vs. wavelength of the view diagram; (**e**) system field curvature and distortion diagram; (**f**) system lateral color diagram.

In the construction of the whole system, multiple objective surfaces, grating diffraction stages, and multiple reflectors are simulated using multiple structures. The optical structure of the microscope objective used is shown in Figure 5a. The optical structure of the microscope objective is in Supplementary Material S2. The simulation results of the reflector-separated light dispersion-compensated 3D microscopy system are shown in Figure 5b. The relationship between RMS and the field of view is shown in Figure 5c. The relationship between RMS and wavelength is shown in Figure 5d. Figure 5e shows the dispersion and field curvature of the system. Figure 5f shows the lateral color of the system. It can be seen that the lateral color of the system is within the diffraction limit.

**Figure 6:**
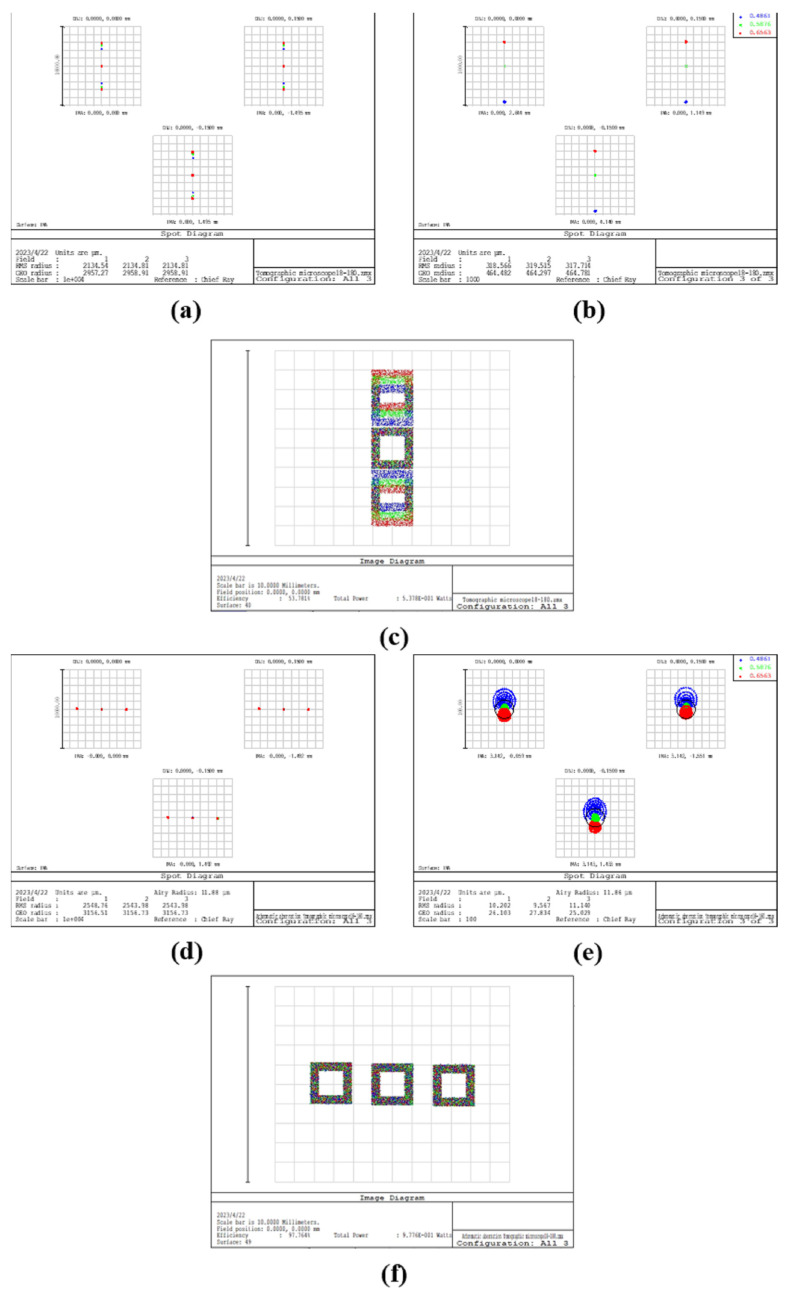

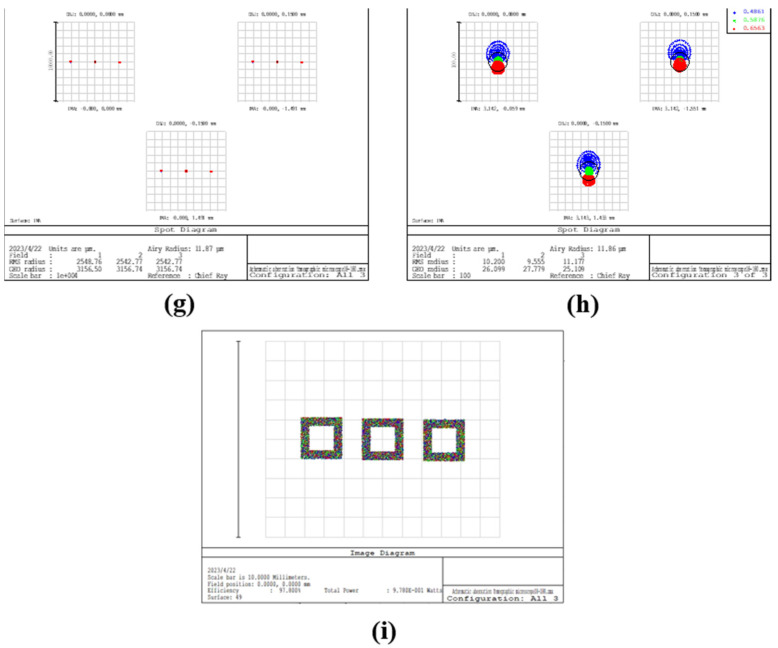
(**a**) Combined spot diagram of three diffraction stages of the normal tomographic microscope system, with different colors representing different wavelengths; (**b**) +1 diffraction stage spot diagram, with different colors representing different wavelengths; (**c**) image simulation results corresponding to +1 diffraction stage; (**d**,**g**) combined spot diagram of the three diffraction stages of the reflector-separated light dispersion-compensated 3D microscopy system, with different colors representing different wavelengths; (**e**,**h**) spot diagram of the +1 diffraction stage of the reflector-separated light dispersion-compensated 3D microscopy system, with different colors representing different wavelengths; (**f**,**i**) image simulation results corresponding to the +1 diffraction stage of the reflector-separated light dispersion-compensated 3D microscopy system.

The reflector-separated light dispersion-compensated 3D microscope system design was completed, and its chromatic aberration correction function was verified. Figure 6 shows the spot diagram and image simulation results for the normal tomographic microscope and two sets of reflector-separated light dispersion-compensated tomographic microscopes, where ($L_{1}$, $L_{2}$) was taken as (60 mm, 50 mm) and (55 mm, 30 mm).

Comparing the three diffraction levels in Figure 6, we can see that the chromatic-aberration-corrected 3D microscopy system shows a reduction in chromatic aberration compared to the normal tomographic microscopy system, with the number of spots at three wavelengths on the detector reducing from seven to three. When comparing with the +1 diffraction-level spot diagram, the center distance of different wavelength spots is changed from 3 mm to 0.1 mm, a reduction of more than 90%. Comparing the image simulation results, we can see that the image of the ordinary tomographic microscope system is formed by the staggered superposition of three wavelength images and that the parts of the three wavelengths that do not overlap have obvious chromatic aberration. In contrast, the image obtained by the separated light dispersion-compensated 3D microscopy system is a clear color image.

## 4. Conclusions

Based on the principle of dispersion compensation and Fourier phase-shift theorem, this paper presents three blazed gratings and reflectors as the separated light dispersion-compensation module of the tomographic system. The separated light dispersion-compensated 3D system and its supporting tube lens were designed. The design results show that the system reduced the chromatic aberration of ordinary tomographic microscopy systems by more than 90%, retaining more wavelengths of light information. In addition, the system had a relatively wide range of color difference compensation element installation positions, reducing the difficulty of dispersion compensation element installation. Using the same substrate for blazed grating and tomographic grating also avoided the effect of prism refractive index errors on chromatic aberration compensation. Overall, the results indicate that the proposed system is effective in reducing chromatic aberration in grating diffraction.

## Figures and Tables

**Table 1:** Partial system parameters.

Parameters	Value
Microscope objective focal length	18 mm
Object-side numerical aperture	0.3
Design wavelength	F, D, C
Tomographic grating center period	2.5 μm
Blazed grating period	2.5 μm
The angle of inclination of the main reflector	21.5°

**Table 2:** The technical parameters of the imaging module.

Parameters	Value
Focal length of the tube lens	180 mm
Half-field of view	2°
Diameter of the pupil	40 mm
MTF	>0.4@144 lp/mm
Detector image element size	3.45 μm
Detector size	8.4 mm × 6.6 mm
Tilt angle between reflectors	±0.5°

## Data Availability

No new data is created.

## Supplementary Materials

The following supporting information can be downloaded at: https://www.mdpi.com/article/10.3390/s23094516/s1, The optical structure of the tube lens and microscopic objective is stored as a Zemax file in the supplementary material. S1: tube lens180; S2: Objective.
